# Latest advances in imaging oxidative stress in cancer

**DOI:** 10.2967/jnumed.120.256974

**Published:** 2021-08-05

**Authors:** Hannah E. Greenwood, Timothy H. Witney

**Affiliations:** 1 School of Biomedical Engineering & Imaging Sciences, King’s College London, London, UK

**Keywords:** ROS, oxidative stress, antioxidant, molecular imaging, MRI, PET, fluorescence

## Abstract

Oxidative stress is the imbalance of harmful reactive oxygen species (ROS) and the action of neutralizing antioxidant mechanisms. If left unchecked, the deleterious effects of oxidative stress results in damage to DNA, proteins, and membranes, ultimately leading to cell death. Tumors are highly proliferative and consequently generate high levels of mitochondrial ROS. To compensate and maintain redox homeostasis, cancer cells upregulate protective antioxidant pathways, which are further amplified in drug-resistant tumors. This review provides an overview of the latest molecular imaging techniques designed to image oxidative stress in cancer. New probes are now able to assess heterogeneous ROS and antioxidant production within tumors and across lesions. Together, the non-invasive imaging of these dynamic processes holds great promise for treatment response monitoring, prediction of drug resistance, and may provide insight into the metastatic potential of tumors.

Cancer cells acquire metabolic adaptations during their transformation which sustains their rapid proliferation, progression, and protection from cell death (1). This ‘metabolic reprogramming’ provides the basis for the clinical imaging and staging of tumors with ^18^F-2-fluoro-2-deoxy-D-glucose (^18^F-FDG) positron emission tomography (PET). The ability to take up glucose and secrete lactate even when oxygen is present (termed aerobic glycolysis) is a key feature of malignancy (2). However, whilst defective mitochondrial respiration was historically thought to accompany aerobic glycolysis, tumors concurrently metabolize glucose through *both* glycolysis *and* the tricarboxylic acid cycle at rates far higher than healthy tissue (3).

Oxidative stress, the imbalance between harmful reactive oxygen species (ROS) production and the cell’s ability to neutralize these reactive intermediates (Fig. 1A), is a common consequence of elevated mitochondrial respiration. Leakage of electrons from complex I and III of the electron transport chain result in the partial reduction of oxygen and the subsequent generation of ROS. These reactive species include hydrogen peroxide (H_2_O_2_), singlet oxygen (^1^O_2_), the hydroxyl radical (·OH), peroxides (O_2_^2−^), and superoxides (O_2_·^−^). Other subcellular regions of ROS generation include peroxisomes (β-oxidation of fatty acids) and the endoplasmic reticulum (protein oxidation), or as by-products of enzymatic reactions by cyclooxygenases, nicotinamide adenine dinucleotide phosphate oxidases, xanthine oxidases, and lipoxygenases (4). Furthermore, multiple components of the tumor-immune microenvironment, such cancer-associated fibroblasts and myeloid-derived suppressor cells, macrophages, and activated T-cells provide an exogenous source of ROS (5).

In conjunction with DNA damage, chemotherapy and radiotherapy produce high levels of oxidative stress in tumors (6). If left unchecked, oxidative stress causes damage to DNA, proteins, and lipids, and ultimately the initiation of cell death. To maintain redox homeostasis and prevent the harmful consequences of oxidative stress, cancer cells upregulate a network of ROS scavenging enzymes and antioxidant pathways (7). As well as generating mitochondrial ROS, cancer metabolism fuels antioxidant production through oxidative pentose phosphate pathway (PPP) generation of reduced nicotinamide adenine dinucleotide phosphate (NADPH) and amino acid metabolism (Fig. 1B). NADPH maintains the antioxidant capacity of thioredoxin reductase and glutathione peroxidase, whereas import of cysteine via system x_C_^-^ is required for glutathione biosynthesis, the body’s most abundant antioxidant (8). In this review we describe the exciting recent advances in the field of oxidative stress imaging and their potential applications.

## Fluorescent Probes

Fluorescence-based imaging systems and probes are widely used for the measurement of a broad spectrum of ROS (9). Elevation of ROS above baseline levels (e.g. following therapeutic intervention) is often assumed to be synonymous with oxidative stress, although in reality oxidative stress can only be inferred from their measurement. A common method to detect multiple forms of ROS is the use of a reduced non-fluorescent dye that once oxidized produces a fluorescent product (switch-on sensors). Hydrocyanines are a class of fluorescent probes that are produced by reducing the iminium cation of commercially available cyanine dyes with NaBH_4_. Upon their oxidation by superoxide and hydroxyl radicals, the original cyanine dye is formed. These dyes fluoresce from 560 to 830 nm and are ionic impermeable moieties, resulting in their intracellular trapping and the generation of contrast (10). Thiophene-bridged hydrocyanine probes overcome some of the limitations of the first-generation probes, which suffer from high autoxidation, low Stokes shifts, and poor stability. Another widely-used switch-on sensor for generalized ROS detection are the CellROX family of compounds (11).

Mitochondria and the plasma membrane are particularly vulnerable to oxidative damage. If left unchecked, oxidative stress results in lipid peroxidation, which can be measured by BODIPY 581/591 C11. Multiple ROS species can oxidize the polyunsaturated butadienyl substituent, resulting in a shift in fluorescent emission from 590 nm to 510 nm. Changes in lipid ROS can subsequently be quantified by measuring the ratio of red to green fluorescence (11).

### Probes For the Selective Imaging of Individual Reactive Species

In addition to assaying oxidative activity in cells, fluorescent probes have been developed for ‘species-specific’ ROS detection, including superoxide (dihydroethidium), hydrogen peroxide (2’,7’-dichlorofluorescin), and singlet oxygen (*trans*-1-(2’-methoxyvinyl)pyrene). Most probes are not truly specific for individual reactive species; rather, they exhibit enhanced selectivity for different ROS. These fluorescent probes rely on varied mechanisms of action to generate contrast. For example, Amplex Red is selectively oxidized by hydrogen peroxide in a reaction mediated by horseradish peroxidase. 2’,7’-dichlorofluorescin (H_2_DCF) is an alternative dye used for the quantitation of intracellular hydrogen peroxide. In its diacetate form, H_2_DCFDA, the nonfluorescent probe, passively diffuses through the cell membrane where it is cleaved by esterases to H_2_DCF, resulting in intracellular trapping. H_2_DCF is then oxidized by hydrogen peroxide to produce 2’,7’-dichlorodihydrofluorescein, which is highly fluorescent. Mitochondrial-specific superoxides can also be visualized by MitoSOX Red, a cationic derivative of dihydroethidium which is electrophoretically taken up into actively respiring mitochondria and fluoresces following its oxidation and subsequent binding to DNA.

### Imaging Glutathione

As the most abundant thiol-containing antioxidant, glutathione is a surrogate marker of cellular antioxidant capacity. Fluorescent dyes monobromobimane and monochlorobimane readily react with low molecular weight thiols, including glutathione, and in doing so form fluorescent adducts. An additional thiol-tracking dye is ThiolTracker™ Violet which is also retained intracellularly through adduct formation and whose fluorescent signal is 10-times greater than bimane compounds (11).

An important consideration is that optical imaging is constrained by overlying tissue both absorbing and causing scatter of the exciting/emitted light. Fluorescence is therefore better suited for cell-based imaging and superficial or intra-operative small animal preclinical work, rather than translational applications.

## Positron Emission Tomography Imaging

Systemic oxidative stress has been assessed in the clinic by measuring oxidized protein and lipids, and serum antioxidants (12). Whilst relatively easy to collect and measure, these biomarkers provide no tissue-specific information which may better-inform any subsequent intervention. Molecular imaging using PET can reveal subtle biological changes that occur both within tumors and across multiple heterogeneous lesions.

### PET Imaging of ROS

The successful application of fluorescent probes for ROS and antioxidant imaging has resulted in the adaptation of these small molecules for PET, often through the incorporation of fluorine-18. ‘Turn-on’ mechanisms following radiotracer oxidation, however, cannot be utilized for the generation of contrast by PET and alternative methods of intracellular trapping are required. Chu *et al*. demonstrated the advantages of radiolabeling the fluorescent dye dihydroethidium with fluorine-18 and showed its ability to measure superoxide production following treatment with doxorubicin in cells grown in culture. Following its oxidation, fluorine-18 labeled dihydroethidium becomes charged and can intercalate DNA, intracellularly trapping the tracer (13). Other fluorescent scaffolds, such as hydrocyanines (14), have also been labeled with fluorine-18 as a method to image oxidative stress *in vivo*. In addition to ROS-sensing fluorophores, chemiluminescent probes based on luminol have been used for ROS detection. Recently, a gallium-labeled luminol derivative (Galuminox) was shown to selectively accumulate in the mitochondria of tumor cells following ROS induction, with ^68^Ga-Galuminox selectively retained in a model of lung inflammation (15). A radiolabeled ascorbate derivative, ^18^F-KS1, is also in the early stages of development for ROS imaging (16).

### Imaging the Tumor Antioxidant Response

**Figure 2:**
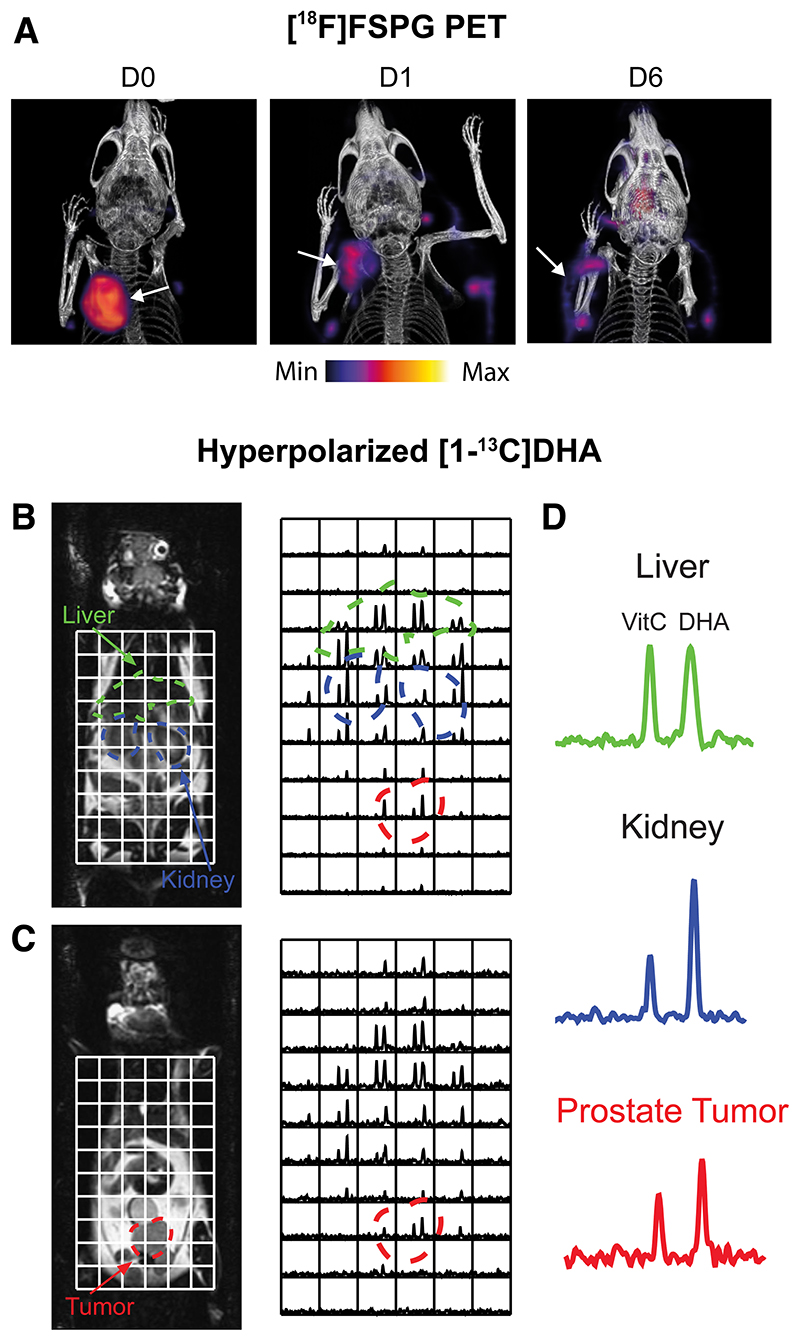
Imaging tumor redox status. (A) Changes in ^18^F-FSPG A2780 ovarian tumor retention following oxidizing Doxil therapy. D0, untreated; D1, 24 h Doxil treatment; D6, 6 days after initiation of Doxil treatment (21). (B, C) Sequential coronal T_2_-weighted images and corresponding ^13^C 3D magnetic resonance spectroscopic images demonstrating distribution of hyperpolarized [1-^13^C]DHA and Vitamin C (VitC) in a TRAMP mouse. Regions of liver, kidney, and prostate tumor are segmented and superimposed on the spectral grid (color-coded dashed lines). (D) Representative ^13^C spectra from liver, kidney, and prostate tumor in a TRAMP mouse (25).

Given the short-lived nature of ROS, imaging the durable downstream consequences of this toxic insult may provide a larger detection window with PET. The transmembrane protein system x_C_^-^ is a heterodimeric transporter that is placed centrally within the cell’s antioxidant system. The role of system x_C_^-^ is to exchange the intracellular amino acid glutamate for the extracellular amino acid cystine. Following cystine’s uptake, it is rapidly reduced to cysteine, the rate limiting precursor for glutathione biosynthesis, placing system x_C_^-^ as a central regulator of antioxidant homeostasis (17). Elevated system x_C_^-^ activity has been exploited by PET imaging tracers such as (4S)-4-(3-^18^F-fluoropropyl)-L-glutamate (^18^F-FSPG) (18), ^18^F-5-fluoro-aminosuberic acid (19), and ^18^F-hGTS13 (20). Tumor retention of ^18^F-FSPG is redox-sensitive, mediated by the concentration gradient of cystine across the plasma membrane. In an animal modal of ovarian cancer, ^18^F-FSPG tumor retention decreased in proportion to the degree of oxidative stress induced by chemotherapy (Fig. 2A) (21).

A consequence of ROS-induced membrane peroxidation is the intracellular production of reactive aldehydes that if left unchecked result in catastrophic DNA damage. Many cancer cells upregulate aldehyde dehydrogenases in response to this oxidative stress, which mediates aldehyde detoxification (22). The enzymatic activity of aldehyde dehydrogenase 1A1 has recently been quantified with a novel substrate-based radiotracer (23). Using a complementary strategy, Kirby *et al.* developed ^18^F-NA_3_BF_3_ for the imaging of total aldehydic load through radiotracer-aldehyde complex formation (24). Together, these tracers may provide insight into oxidative stress-mediated lipid peroxidation during anti-cancer therapy.

## Magnetic Resonance Imaging

Several paramagnetic MRI contrast agents have been developed to probe the redox balance of cells and tissues. Stable nitroxide free radicals are cell-permeable reporters of intracellular antioxidant availability, undergoing one-electron transfer reactions to produce hydroxylamines. The single unpaired electron of nitroxides provides T_1_-contrast which disappears upon their reduction, the rate of which is dependent on ROS-scavenging systems (26). Nitroxide relaxivity, however, is 20 times less than Gd^3+^ and contrast is quickly lost following administration. Alternative MRI contrast agents based around activatable paramagnetic complexes have subsequently been developed to overcome these limitations. The oxidation state of both Mn^3+/2+^ (27) and Fe^3+/2+^ complexes (28) alter the intrinsic relaxation properties of MRI probes, enabling a non-invasive measure of cellular redox status.

### Hyperpolarized Magnetic Resonance Spectroscopic Imaging

Dynamic Nuclear Polarization is an emerging technique that increases the sensitivity of magnetic resonance experiments by >10,000 times, allowing dynamic imaging of administered ^13^C-labeled substrates and their metabolic products *in vivo* (29). Flux through the PPP has been estimated using this technique through the conversion of U-^2^H,U-^13^C-glucose to the PPP intermediate 6-phosphogluconate (30). PPP metabolic activity is upregulated in cancer, which generates NADPH to maintain the antioxidant capacity of cells. However, the short polarization lifetime of uniformly-labeled glucose at relatively low levels (~15%), along with overlapping ^13^C resonances of 6-phosphogluconate and 3‐phosphoglycerate (a glycolytic intermediate) are challenges which currently restricts the widespread use of U-^2^H,U-^13^C-glucose. Alternatively, hyperpolarized 1-^13^C-dehydroascorbic acid (1-^13^C-DHA), the oxidized form of ascorbic acid (Vitamin C), has been used to probe tumor redox potential (31). After its uptake by the facilitative glucose transporters, hyperpolarized 1-^13^C-DHA was rapidly converted to 1-^13^C-Vitamin C in lymphoma (31) and prostate tumors (Fig. 2B-D) (25), the rate of which was determined by the levels of both glutathione and NADPH (32). Despite the promise of [1-^13^C]DHA to assess total tumor antioxidant capacity, administration of 10 mg/kg DHA to tumor-bearing mice resulted in transient respiratory arrest and cardiac depression (32). Optimization of dosing regimens and a greater understanding of DHA toxicity are therefore a prerequisite for clinical imaging with 1-^13^C-DHA.

An important consideration for all MRI-based redox probes is the requirement of high mass doses of contrast agent. Given that these agents are frequently either stable radicals or potent radical scavengers, redox-active MRI probes may also perturb the system that they are measuring, possibly accounting for DHA-induced toxicity.

## Applications and Future Perspectives

As we have illustrated, several well-characterized imaging agents have shown promise for the non-invasive imaging of oxidative stress in animal models of cancer. Given that ROS are typically short-lived (T_1/2_ of 10^-9^ s for ·OH to 10^-3^ s for H_2_O_2_ (33)) and encompass a variety of different reactive molecules, frequently at low concentrations, imaging ROS dynamics is a challenging proposition. The cellular antioxidant response to these insults, however, persists on a timescale and magnitude that permits its measurement by medical imaging techniques. If clinically adopted, several applications exist for oxidative stress imaging that could impact disease outcomes.

### Response Monitoring

In conjunction with DNA damage, chemotherapies and ionizing radiation produce high levels of oxidative stress in tumors, with cell death induced in those sensitive to treatment (6). Consequently, redox imaging agents have the potential to assess the efficacy of a wide-range of therapies that converge with the induction of oxidative stress. In a recent proof-of-principle study, the tumor antioxidant response to doxorubicin was shown to be an earlier maker than both ^18^F-FDG and tumor volume (21). Furthermore, the imaging window for the measurement of tumor antioxidant response is not limited by a temporally unstable marker (*e.g.* cell death) (34). Additionally, a number of therapies have been developed whose primary mechanism of action is the induction of lipid ROS and concurrent membrane peroxidation (35). Redox imaging probes may therefore play an important role for the monitoring of response to these novel agents.

### Prediction of Drug Resistance

Elevated antioxidant capacity and the ability to buffer oxidative stress is a hallmark of drug resistant cancer (36). A non-invasive measure of drug resistance will facilitate early intervention, allowing the clinician to adapt the treatment regimen, with the potential to improve patient outcomes. For widespread utility, the imaging biomarker ideally should: 1. be causal to drug resistance; 2. be tumor-specific; 3. result in a positive imaging signal; 4. be generalizable to multiple drugs with different mechanisms of action; 5. have expression that is independent of other factors/conditions; and 6. require a single imaging scan. To-date, ^18^F-FSPG imaging of system x_C_^-^ activity has proven to be a good surrogate marker of drug resistance in animal models of ovarian cancer, reporting on the elevated glutathione found in these tumors (37). Further work, however, is needed to determine whether ^18^F-FSPG is a robust marker of drug resistance for multiple cancer types with discrete driver mutations.

### Metastases

Tumor cells experience substantial oxidative stress when they detach from the extracellular matrix and enter the circulation. Anoikis, a form of programmed cell death following loss of anchorage, frequently follows intravasation and restricts the metastatic capabilities of tumor cells (38). The oxidative environment of the bloodstream further limits metastatic efficiency. In anoikis-resistant cells, PPP-generated NADPH mitigates the ROS that accompanies loss of attachment to permit cell survival (39). Suppressing oxidative stress by increasing endogenous and exogenous antioxidant availability *in vivo* further promotes metastasis in multiple models of cancer (1). Consequently, by imaging tumor antioxidant capacity before membrane detachment, it may therefore be possible to determine the metastatic potential of primary tumors.

## Conclusion

The spatiotemporal assessment of the tumor redox microenvironment *in vivo* has the potential to inform cancer progression, therapeutic response, and metastatic potential. The preclinical development of non-invasive MRI and PET imaging agents is set to revolutionize our understanding of these dynamic processes, complementing the existing arsenal of ROS-sensing fluorophores. Clinical validation of the existing imaging agents, however, is still to be performed, along with the assessment of their prognostic utility. Additional redox-active probes are also required whose tumor retention is sensitive to the balance between ROS and the antioxidant response, rather than simple turn-on signal. With ^18^F-FSPG already trialed in patients, there is a reasonable expectation that the first mechanistic clinical studies with this radiotracer will be performed in the near future.

## Disclosure

This study was funded through a Wellcome Trust Senior Research Fellowship (220221/Z/20/Z) to T.H.W and supported by Wellcome/EPSRC Centre for Medical Engineering (WT203148/Z/16/Z) to H.E.G. As this research was funded by the Wellcome Trust and for the purpose of open access, the author has applied a CC BY public copyright license to any Author Accepted Manuscript version arising from this submission. No potential conflicts of interest relevant to this article exist.

## Figures and Tables

**Figure 1:**
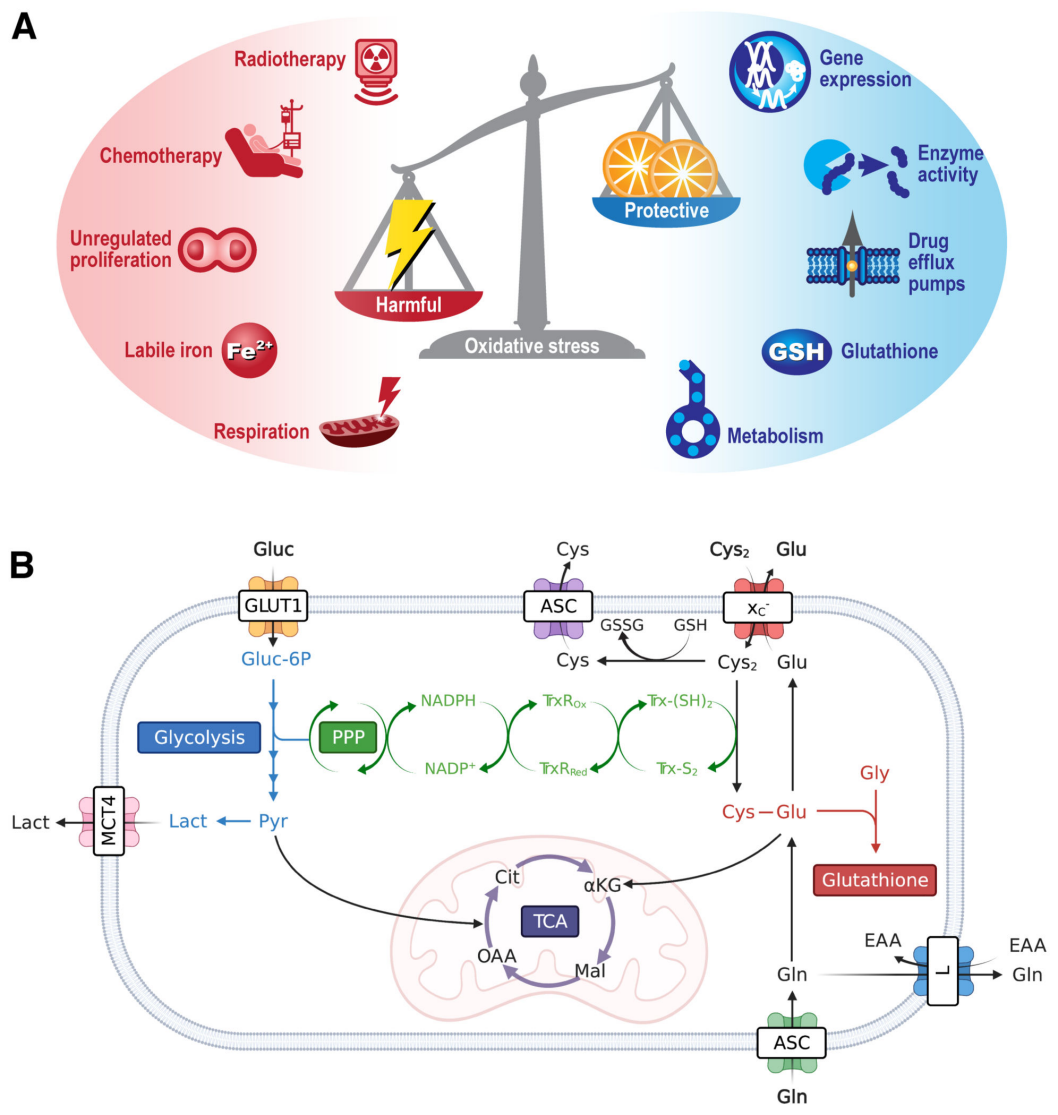
Mediators and protective mechanisms that regulate oxidative stress. (A) Oxidative stress is an imbalance between harmful ROS and neutralizing antioxidants. ROS can be formed either by intrinsic or extrinsic factors, with a network of intracellular free radical scavenger systems designed to maintain redox homeostasis and protect against cellular damage. (B) Metabolism is a key regulator of intracellular antioxidants NAPH, glutathione, and the thioredoxin pathway. For clarity, the tricarboxylic acid cycle and glycolysis have been abbreviated. PPP, pentose phosphate pathway; TCA, tricarboxylic acid cycle; ASC, alanine/serine/cysteine transporter subfamily; GLUT1, Glucose transporter 1; L, system L amino acid transporter; MCT4, monocarboxylate transporter 4; αKG, α-ketoglutarate; Cit, citrate; Cys, cysteine; Cys_2_, cystine; EAA, essential amino acids; Glu, glutamate; Gln, glutamine; Gly, Glycine; Gluc, glucose; Gluc-6P, glucose 6-phosphate; GSH, glutathione; GSSG, oxidized glutathione; Lact, lactate; Mal, malate; NADPH, reduced nicotinamide adenine dinucleotide phosphate; NADP^+^, nicotinamide adenine dinucleotide phosphate; OAA, oxaloacetate; Pyr, pyruvate; TrxR_ox_, oxidized thioredoxin reductase; TrxR_Red_, reduced thioredoxin reductase; Trx-(S_2_), thioredoxin-disulfide reductase; Trx-(SH_2_), thioredoxin-dithiol reductase; x_C_^-^, system x_C_^-^.
